# CSI Feedback Model Based on Multi-Source Characterization in FDD Systems

**DOI:** 10.3390/s23198139

**Published:** 2023-09-28

**Authors:** Fei Pan, Xiaoyu Zhao, Boda Zhang, Pengjun Xiang, Mengdie Hu, Xuesong Gao

**Affiliations:** 1 College of Information Engineering, Sichuan Agricultural University, Ya’an 625014, China; 2 Ya’an Digital Agricultural Engineering Technology Research Center, Ya’an 625014, China; 3 College of Resources, Sichuan Agricultural University, Chengdu 625099, China

**Keywords:** CSI feedback, neural network, FDD, deep learning, wireless communication

## Abstract

In wireless communication, to fully utilize the spectrum and energy efficiency of the system, it is necessary to obtain the channel state information (CSI) of the link. However, in Frequency Division Duplexing (FDD) systems, CSI feedback wastes part of the spectrum resources. In order to save spectrum resources, the CSI needs to be compressed. However, many current deep-learning algorithms have complex structures and a large number of model parameters. When the computational and storage resources are limited, the large number of model parameters will decrease the accuracy of CSI feedback, which cannot meet the application requirements. In this paper, we propose a neural network-based CSI feedback model, Mix_Multi_TransNet, which considers both the spatial characteristics and temporal sequence of the channel, aiming to provide higher feedback accuracy while reducing the number of model parameters. Through experiments, it is found that Mix_Multi_TransNet achieves higher accuracy than the traditional CSI feedback network in both indoor and outdoor scenes. In the indoor scene, the NMSE gains of Mix_Multi_TransNet are 4.06 dB, 4.92 dB, 4.82 dB, and 6.47 dB for compression ratio *η* = 1/8, 1/16, 1/32, 1/64, respectively. In the outdoor scene, the NMSE gains of Mix_Multi_TransNet are 3.63 dB, 6.24 dB, 4.71 dB, 4.60 dB, and 2.93 dB for compression ratio *η* = 1/4, 1/8, 1/16, 1/32, 1/64, respectively.

## 1. Introduction

### 1.1. Background and Motivations

With the development of mobile communication technology, the need for high-precision channel feedback is becoming increasingly urgent. In a large-scale Multi-Input Multi-Output (MIMO) system, the Base Station (BS) is configured with a large number of antennas to fully utilize spatial diversity and spatial multiplexing to increase the channel capacity [1]. As the amount of transmitted data increases, the channel becomes congested. We can use reconfigurable intelligent surfaces (RISs) for safe and efficient effects. RISs act as a relay to strengthen the signal strength and provide energy for the subsequent signal transmission [2,3]. RISs mainly serve as a relay to enhance the transmission energy. Beamforming is required to concentrate the signal energy on specific User Equipment (UE) and achieve less interference and leakage at a high signal-noise ratio (SNR) while ensuring the transmission [4]. One of the keys to achieving beamforming is to have accurate downlink Channel State Information (CSI). Usually, in Time Division Duplexing (TDD) systems where uplink data and downlink data can be transmitted at the same frequency point, the uplink and downlink channels are reciprocal, i.e., the UE can back-propagate the uplink channel through the downlink channel and, thus, does not have to spend many resources to implement the CSI estimation [5].

In recent years, Frequency Division Duplexing (FDD) systems have gained more popularity due to their higher spectrum utilization [6]. In FDD, on the other hand, the uplink and downlink channels use different frequency points. Therefore, channel reciprocity cannot be utilized. In this case, the downlink channel state information can only be estimated at the UE side and then fed back to the BS. However, this feedback step requires additional resource overheads. As the number of antennas increases, this overhead grows quadratically and even reduces the competitive advantage of MIMO systems [7].

Currently, the mainstream methods of CSI feedback are categorized into three types: codebook-based CSI feedback, Compressed Sensing (CS)-based CSI feedback, and Deep Learning (DL)-based CSI feedback.

A codebook-based CSI feedback study requires a codebook known at the BSand UE sides. During feedback, the CSI is first converted into codebook information for compression purposes and then the codebook information is fed back to the BS through the feedback link. At the BS end, the CSI is recovered by comparing the codebook information. Literature [8] proposes an adaptive codebook that adapts the codebook to any variable by deriving the angular distribution between the channel vector and the Line of Sight (LoS) path component to share an identical codebook at the transmitter and receiver. However, as the amount of data increases, the codebook becomes more extensive and the computational complexity grows exponentially concerning the codebook size. To overcome the complexity problem, literature [9] proposes an environmental knowledge codebook CSI-based feedback framework. The framework enhances the learning of environmental knowledge by introducing a simple neural network, improving the output of the codebook-based channel feedback. This scheme reduces the computational complexity to a certain extent, but the codebook size does not change and the cost of searching the codebook is still high.

In order to solve the problem of the high time cost of codebook-based algorithms, researchers have applied CS methods in CSI feedback. The CSI feedback method based on 1-Bit CS acquires CSI with a higher accuracy without occupying uplink bandwidth resources alone [10]. However, this method can only be realized at smaller compression ratios. Literature [11] utilizes the correlation between the received and transmitted signals for iterative estimation. This method utilizes the signal’s sparsity to reduce the amount of sampled data, thus achieving compression. However, the algorithm requires strict sparsity of the channel and consumes a lot of computational resources and time as the number of iterations of the CS algorithm increases.

With the development and broad application of deep learning in many fields, deep learning has also been introduced into wireless communications [12,13,14,15,16]. Meanwhile, since DL supports end-to-end communication systems, the sender and receiver can use neural network representations in self-coding [17,18,19]. Therefore, in order to overcome the problems of high computational resources and the high time cost of codebook-based and CS-based CSI feedback, literature [20] draws on the idea of the self-coding form of neural networks and applies DL to the CSI feedback model CsiNet, where the downlink CSI is regarded as a special kind of “image” and the model is constructed as a self-coding neural network. The model is constructed as a self-coding neural network. CsiNet compresses and recovers the CSI through a simple self-encoder neural network structure using convolution, proving the feasibility of a neural network for CSI feedback.

Moreover, CsiNet outperforms traditional codebook and CS methods at all compression ratios. After that, most DL-based feedback methods borrowed from CsiNet networks, e.g., CsiNet+ [21], inherited most of the architecture of CsiNet. They learned more spatial features by updating the convolutional kernel using a parallel multi-rate compression method, improving network performance and feedback accuracy. However, the algorithm only focuses and trains on the spatial features of the CSI, ignoring the temporal nature of CSI. JC-ResNet [22] uses a residual network, resulting in a better channel feature transfer. However, the residual connection may cause irrelevant features to be passed on, thus interfering with the learning process of the model. CRNet [23] employs a multi-channel convolutional path to learn the channel spatial features of different receptive fields, and the different receptive fields compensate for each other to improve the network’s performance. CRNet increases the feedback accuracy to a certain extent, but it singularly focuses on the spatial features of the CSI and similarly neglects the temporal nature of CSI. CLNet [24] weights the complex signals by using the correlation between real and imaginary parts after dividing the complex signals into real and imaginary parts. Compared to other algorithms, CLNet is trained with a relatively simple network structure, which reduces the information loss problem caused by complex splitting and complexity. However, the method also singularly considers the spatial features of the CSI and ignores the temporal nature of CSI. CsiNet-LSTM [25] and Attention-CSI [26] gather an increased temporal nature from the training samples by introducing LSTM. Although both networks focus on the temporal nature of CSI to ensure some feedback accuracy, they ignore the spatial features of CSI. STNet [27] proposes a spatially divisible attention mechanism, which mainly refers to the attention mechanism part of Transformer [28], using local grouping self-attention and global sampling attention. The method improves the performance of the network with some increase in complexity. The algorithm pays some attention to both the spatial characteristics of CSI and the temporal order of the CSI but does not balance the feedback accuracy of CSI.

### 1.2. Contributions

The above networks have paid attention to their spatial characteristics or temporal properties by analyzing the downlink CSI features. However, the networks consider them only from a single part, ignoring the influence between the two factors, or the feedback accuracy is too low. When the network only thinks of the spatial characteristics, the network breaks the connection between the elements in the downlink CSI, which affects the final feedback accuracy. When the network only finds from the temporal nature, the feedback matrix is not sufficiently compressed, which significantly increases the overhead cost of the feedback. The CSI feedback accuracy is improved by paying attention to and learning the spatial characteristics and timing of the downlink CSI. In this study, the Mix_Multi_TransNet network is proposed to consider both the spatial factors and temporality of the downlink CSI, and its main work is summarized as follows:

(1) Use dual-path neural networks. Path1 and Path2 learn the channel matrix’s spatial features and temporal properties, respectively. Path1 adopts multiple sensory fields to understand the channel matrix’s spatial characteristics comprehensively, and Path2 combines the Transformer Attention Mechanism with Convolutional Neural Networks to thoroughly learn the temporal properties of the channel information.
(2) Path1 uses multiple sensory fields; Path2 uses the Transformer attention mechanism combined with a convolutional neural network to ensure the learning of temporal relationships while reducing model parameters. The two path feature representations are eventually fused to improve the model’s performance, robustness, and generalization ability.

### 1.3. Paper Organization

The article consists of five parts in total. This chapter focuses on the background of this study and the innovations and contributions of our entire study; the second part mainly introduces our system model; the third part mainly introduces the design of the Mix_Multi_TransNet network; the fourth part is our experimental results and comparisons; and the fifth part is our conclusion.

## 2. System Model

**Figure 1:**
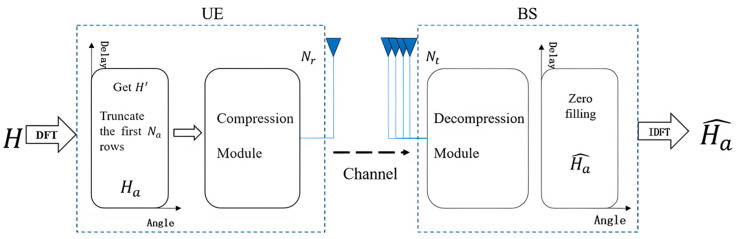
System Model Diagram.

In the FDD system, the BS have $N_{t}$-transmitting antennas and the UE have $N_{r}$ antennas, $N_{t}\geq N_{r}$. For simplicity, this study lets $N_{r}=1$. An Orthogonal Frequency Division Multiplexing (OFDM) system with $N_{c}$ subcarriers is used, and the system model is shown in Figure 1.

The received signal $y\in C^{N_{c}\times 1}$ can be expressed as:

(1) y=Ax+z

where $x\in C^{N_{c}\times 1}$ denotes the transmitted symbol vector in one OFDM cycle and $z\in C^{N_{c}\times 1}$ is the additive noise vector. The diagonal matrix $A=diag(h_{1}^{H}p_{1},\ldots ,h_{n}^{H}p_{n})$, ${n=N}_{c}$, where $h_{i}\in C^{N_{t}\times 1}$ and $p_{i}\in C^{N_{t}\times 1}$, denotes the downlink channel response vector and beamforming precoding vector, $i\in \{1,\ldots ,N_{c}\}$, respectively.

In order to obtain the beamforming vectors, it is necessary to obtain the corresponding $h_{i}$ at the base station. Define the downlink channel matrix $H\epsilon C^{N_{c}\times N_{t}}$. The matrix contains $N_{c}\times N_{t}$ elements, each of which includes information about the real and imaginary parts of the CSI, proportional to the number of antennas. In the case of massive MIMO, $N_{c}\times N_{t}$ will be a vast number, which is a huge challenge for data processing. Since the channel matrix is sparse in the angular time-delay domain, H can be transformed from the spatial frequency domain to the angular time-delay domain using the two-dimensional discrete Fourier transform (DFT), as shown in Equation (2).

(2) $$H^{’}=F_{c}HF_{t}^{H}$$

where $F_{c}$ and $F_{t}$ denote DFT matrices of size $N_{c}\times N_{c}$ and $N_{t}\times N_{t}$, respectively. For each element in the angular delay domain matrix, $H^{’}\epsilon C^{N_{c}\times N_{t}}$ corresponds to a path delay with a certain angle of arrival (AoA). Only the first $N_{a}$ rows of $H^{’}$ obtained after two-dimensional discrete Fourier transform (DFT) contain helpful information, and the rest of the rows indicate paths with considerable propagation delays, so the elements are almost zero. Therefore, only the first $N_{a}$ is truncated to obtain the matrix $H_{a}\epsilon C^{N_{a}\times N_{t}}$ representing the downlink channel matrix.

By truncating $H^{’}$, the channel matrix is downscaled. However, $H_{a}$ is still a matrix with extensive data elements. Therefore, further dimensionality reduction is needed. The neural network can handle the problem of exaggerated dimensions and uneven feature distribution of $H_{a}$ very well. Therefore, $H_{a}$ can be inputted into the encoder part of the UE consisting of neural networks, and the encoder can obtain codeword υ according to the compression ratio η:

(3) $$\upsilon =F_{\xi }(H_{a},\;\Theta_{\xi })$$

where $F_{\xi }(\cdot )$ denotes the compression process and $\Theta_{\xi }$ denotes a set of parameters of the encoder.

After the feedback link is sent to the BS, υ is recovered in the decoder part of the BS. Like the encoder, the decoder also consists of a neural network. The recovery process of $H_{a}$ can be represented as follows:

(4) $$\hat{H_{a}}=F_{R}(\upsilon ,\;\Theta_{R})$$

where $F_{R}(\cdot )$ denotes the recovery process and $\Theta_{R}$ denotes a set of parameters of the decoder. The $\hat{H_{a}}$ obtained after recovery is padded for its zero values and then undergoes a two-dimensional discrete Fourier inverse transform (IDFT) to obtain $\tilde{H}$.

Combining Equations (3) and (4) and using the mean square error as a metric, the entire compression and recovery process can be expressed as:

(5) $$\left(\hat{F_{\xi }},\hat{\Theta_{\xi }}\right)=\underset{F_{\xi },F_{R}}{\mathrm{argmin}}{\left\|H_{a}-F_{R}(F_{\xi }\left(H_{a},\;\Theta_{\xi }\right),\Theta_{R})\right\|}_{2}^{2}$$

It is assumed that the uplink is in an ideal state, i.e., there is no loss in υ obtained from the encoder processing and then passed through the uplink to the decoder and recovered at the decoder. Therefore, the main objective of this study is to train and design the network for $\Theta_{\xi }$ and $\Theta_{R}$.

## 3. Mix_Multi_TransNet Design

**Figure 2:**
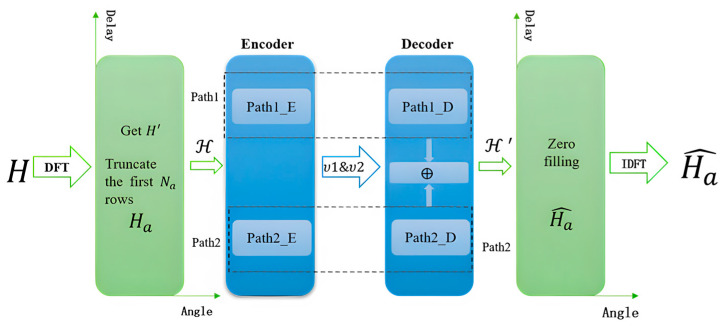
Network Modeling Process.

This section describes the design and principles of the Mix_Multi_TransNet network and its key components. The overall architecture of Mix_Multi_TransNet is shown in Figure 2. Mix_Multi_TransNet is an encoder-decoder framework divided into two paths with four embedded modules to solve the CSI feedback problem.

### 3.1. Network Modeling Processes

As shown in Figure 2, the whole network is divided into two parts: the encoder and the decoder. Before entering the encoder, firstly, the two-dimensional discrete Fourier transform of H is utilized to obtain $H^{’}$ using Equation (2), and then truncation is performed to obtain the complex matrix $H_{a}$. Then, the complex matrix $H_{a}\epsilon C^{N_{a}\times N_{t}}$ is directly divided into real and imaginary parts:

(6) $$Re\left(H_{a}\right)=a\;;Im\left(H_{a}\right)=b$$

a denotes the real part of the complex number and b denotes the imaginary part of the complex number; then, the resulting real and imaginary parts are turned into a $2N_{a}\times N_{t}$ new matrix:

(7) $$H_{\left[k\right][j]}=\left\{\begin{array}{c} Re\left({H_{a}}_{\left[i\right][j]}\right),0\leq k<N_{a}\\ Im\left({H_{a}}_{\left[i\right][j]}\right),N_{a}\leq k<2N_{a} \end{array}\right.$$

where 0≤i<Na,0≤j<Nt,0≤k<2Na, and i=k or i=k−Na. The new matrix H is taken as input into both Path1 and Path2 of the encoder, and the whole processing in the encoder is conducted as shown in Equation (6); finally, υ1&υ2 is obtained after passing through Path1_E and Path2_E. In the decoder, υ1&υ2 is input into Path1 and Path2, corresponding to the decoder, respectively. υ1&υ2 is obtained after passing through Path1_D and Path2_ D processing and ${\upsilon 1}^{’}\&{\upsilon 2}^{’}$ is obtained; then, the two matrices of ${\upsilon 1}^{’}\&{\upsilon 2}^{’}$ are fused:

(8) $$H^{’}={\upsilon 1}^{’}⨁{\upsilon 2}^{’}$$

The obtained $H^{’}$ is recovered as a complex matrix $\hat{H_{a}}$. The entire processing in the decoder is shown in Equation (4).

### 3.2. Path1

**Figure 3:**
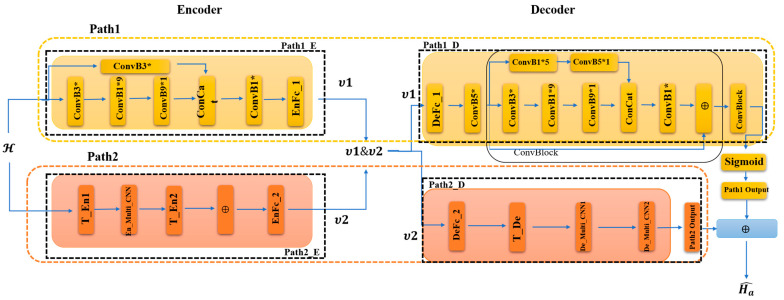
Encoder and Decoder. ‘*’ denotes the convolution dimension hyphen, e.g., 3* denotes a 2D kernel of size 3×3; ‘⨁’ indicates the addition of two matrices.

A detailed description of the encoder part and decoder part of the network is given, as shown in Figure 3. From Figure 3, it can be seen that the whole self-encoding network is divided into two paths, Path1 and Path2. Path1 consists of Path1_E and Path1_D, denoted as the encoder and decoder part, respectively.

In order to thoroughly learn the spatial features of the channel matrix and to reduce the network parameters, Path1_E and Path1_D use a multidimensional pure convolutional structure. Due to the sparsity of the channel matrix in the angular delay domain, the distribution of the channel state information is still unevenly distributed after the interception of the former $N_{a}$ rows. The convolution structure in Path1_E adopts two two-dimensional convolution operations with two different convolution sizes and a number of convolutions to enhance the effect of learning the features of the channel matrix, reducing the network parameters and enabling fast training. In Path1_E, the first path consists of ConvB3*, ConvB1*9, and ConvB9*1, indicating three convolution sizes of 3*3, 1*9, and 9*1 structures, respectively. Each 2D convolution is as in Equation (9):

(9) $$Y_{2D}=Conv2D\left(Y_{in},W_{in}\right)$$

$Y_{in}$ denotes the matrix of the input convolution and $W_{in}$ denotes the corresponding convolution weights. In this path, $H_{a}$ first passes through the ConvB3* module and the convolution weight matrix has a size of 3*3. Then, the result is fed into ConvB1*9 and the corresponding convolution weight matrix has a size of 1*9. Finally, after ConvB9*1, the convolution weight matrix has a size of 9*1. After ConvB3*, ConvB1*9, and ConvB9*1, we get the feature matrix $Y_{p1}\_E1$ of the first large convolution path. Large convolution operation can increase the sensory field and obtain more null domain information of the channel matrix. However, in order to compensate for the blurring effect of feature information brought by the large convolution operation and to improve the local and fine-grained feature-learning effect, the ConvB3* module of the other path in Path1_E uses the two-dimensional convolution with a convolution size of 3*3. It performs the same operation as in Equation (9) for $H_{a}$ to obtain $Y_{p1}\_E2$. Then, the two paths $Y_{p1}\_E1$ and $Y_{p1}\_E2$, are subjected to the Concat operation:

(10) $$Y_{p1}\_E=Concat\left(\left(Y_{p1}\_E1,Y_{p1}\_E2\right),\;dim=1\right)$$

The resulting two feature matrices are fused with features in the dimension dim=1, and then a 1*1 convolution operation, as in Equation (9), is performed to reduce the network parameters. Note that a batch normalization is performed after each convolution operation:

(11) Y_norm=((Y_i−mean(Y_i))/sqrt(var(Y_i)+ep))*g+b

Here, mean(Y_i) and var(Y_i) denote the mean and variance of the feature matrix Y_i on each channel, ep is a tiny constant used to avoid division by zero, and g and b are the learnable scaling factor and bias term, respectively. The feature matrix Y_i is the result after each convolution. After batch normalization, the training speed of the network is increased, the gradient propagation is improved, and the model’s generalization ability is also improved. The activation function LeakyReLU is then used after batch normalization to provide nonlinearity. Then, after a dimensional change:

(12) $$Y_{view}={Y_{a}}^{’}.view(n,-1)$$

${Y_{a}}^{’}$ denotes the matrix obtained after the activation function. Finally, the obtained sequence data $Y_{view}$ is passed through the fully connected layer EnFc_1:

(13) $$\upsilon 1=Y_{view}{\cdot W}_{view}+B_{view}$$

Here, $W_{view}$ is a weight matrix of shape $\left(total_{size},\frac{total_{size}}{\eta }\right)$ and $B_{view}$ is a bias term of shape $\left(\frac{total_{size}}{\eta },\right)$. The final result obtained from Path1_E is compressed according to the compression ratio η to υ1.

The same 2D convolution operation with different convolution kernel sizes and number of convolutions is used in Path1_D. Path1_D mainly follows the CRBlock module in CRNet [21]. In this way, to recover the channel matrix accurately and efficiently, in Path1_D, it first goes through the fully connected layer DeFc_1 to restore υ1 to the sequence size before compression:

(14) $${\upsilon 1}_{D}=\upsilon 1\cdot W_{D}+B_{D}$$

$W_{D}$ is a weight matrix of shape $\left(\frac{total_{size}}{\eta },total_{size}\right)$ and $B_{D}$ is a shape $\left(total_{size}\;,\right)$. It is restored to the original size, and then goes through the ConvB5* module, which represents a two-dimensional convolution with a convolution size of 5*5, computed as in Equation (9), and keeps the result:

(15) $$\bar{{\upsilon 1}_{D}}=I(Conv2D\left({\upsilon 1}_{D},{W1}_{D}\right))$$

Here, I(⋅) makes the reservation of the calculation result. Then, the obtained result $\bar{{\upsilon 1}_{D}}$ is fed into two convolution paths, in which the first convolution path consists of three parts, ConvB3*, ConvB1*9, ConvB9*1, and each convolution operation is calculated as in Equation (10). After the first convolution path, we get $Y_{p1}\_D1$. The second convolution path consists of ConvB1*5, ConvB5*1, and each convolution operation is calculated as in Equation (9) after the first convolution path to get $Y_{p1}\_D2$, and then the two paths get $Y_{p1}\_D1$ and $Y_{p1}\_D2$ for the Concat operation, as in Equation (16):

(16) $$Y_{p1}\_D=Concat\left(\left(Y_{p1}\_D1,Y_{p1}\_D2\right),dim=1\right)$$

The two feature matrices obtained are fused with features in the dimension dim=1, and then a 1*1 convolution operation is performed as in Equation (9) to reduce the network parameters. Note that after each convolution operation, batch normalization is performed, computed as in Equation (11), and then the activation function LeakyReLU is used to provide the nonlinearity to obtain ${{\upsilon 1}_{D}}^{’}$. Here, a residual join is made between ${{\upsilon 1}_{D}}^{’}$ and the previously retained $\bar{{\upsilon 1}_{D}}$ in order to better transfer the features:

(17) $$\bar{\upsilon 1}=F1\left(\bar{{\upsilon 1}_{D}}\right)+{{\upsilon 1}_{D}}^{’}$$

$F1\left(\cdot \right)$ After denoting the ConvB5* module, a series of operations are obtained for $\bar{{\upsilon 1}_{D}}$. We summarize operations as the ConvBlock module, as shown in Figure 3. Subsequently, the obtained $\bar{\upsilon 1}$ is fed into the next ConvBlock module to obtain $\bar{\upsilon 2}$. Finally, using the Sigmoid function, the output is mapped to [0,1] to obtain the output ${\upsilon 1}^{’}$ of Path1.

### 3.3. Path2

**Figure 4:**
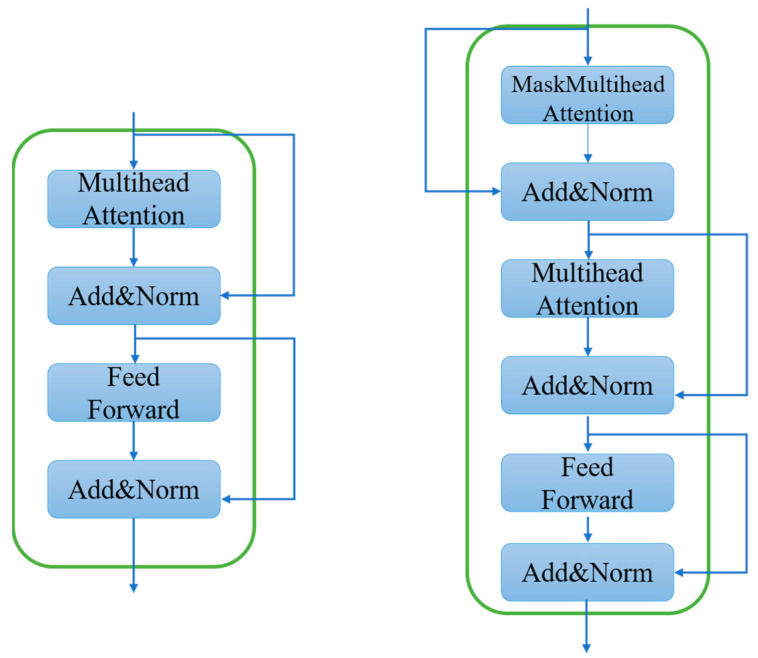
T_En (**Left**) and T_De (**Right**).

Path2 also consists of two parts, Path2_E and Path2_D, which are denoted as the encoder part and decoder part, respectively. Different from Path1, this path mainly focuses on and learns the temporal nature of the CSI to compensate for the shortcoming of Pat1, which only focuses on the spatial features. Path2_E consists of T_EN1, En_Multi_CNN, T_EN2, and EnFc_2. Among them, T_EN1 and T_EN2 structures are shown on the left of Figure 4, respectively, adopting Transformer’s encoder layer structure. Path2_D consists of DeFc_2, T_De, De_Multi_CNN1, and De_Multi_CNN2, of which the T_De structure is shown on the right of Figure 4, adopting Transformer’s decoder layer structure.

First, we will explain how T_En1, T_En2, and T_De work. The input to Path2_En is a complex split with dimensional changes to obtain a new matrix, H. H is first fed into the T_En1 module, which employs the decoder layer of the Transformer. H passes through the multiattendance module, through three separate linear layers. These three independent linear layers will have three outputs, computed as follows:

(18) $$Q_{n}=HW_{n}^{Q},K_{n}=HW_{n}^{K},V_{n}=HW_{n}^{V}$$

where $Q_{n}$, $K_{n}$, and $V_{n}$ represent the outputs of the three linear layers, respectively. $W_{n}^{Q}$, $W_{n}^{K}$, and $W_{n}^{V}$ represent the weights of the three linear layers on the *n*th head of the multi-head attention module, respectively, and then the attention score matrix is calculated:

(19) $$Atten_{score}=Softmax(\frac{Q_{n}K_{n}^{T}}{\sqrt{d}})\;V_{n}$$

where $Q_{n}K_{n}^{T}$ is calculated to derive the degree of correlation between each element of the matrix. In order to eliminate the $Q_{n}$ multiplied by $K_{n}$, the gradient is prevented from vanishing by eliminating the change in magnitude brought about by multiplying the matrix by the K-transpose matrix. After transposing the matrix, divide by $\sqrt{d}$, where d denotes the dimension of the matrix. Then, after the Softmax(⋅) function is normalized, finally, multiply by the matrix $V_{n}$ to obtain the attention score matrix. Then, after residual joining and layer normalization, the processing result is sent to the feed-forward layer and, finally, after one more residual joining and layer normalization to obtain the result of the T_En1 part, the working principle of T_En2 is the same as that of T_En1, but the difference is that the input of T_En2 becomes the processing result of the En_Multi_CNN part.

The corresponding T_De uses Transformer’s decoder layer structure, which works similarly to the encoder layer with the difference that the decoder part uses a masked multi-head attention mechanism. Unlike the multi-head attention mechanism, the masked multi-head attention mechanism uses a whole new layer of weights to represent the criticality of each part of the feature data. It uses a masking mechanism to prevent label leakage. The T_De1 section first undergoes processing by the masked multi-head attention mechanism, followed by feeding the results into residual concatenation and layer normalization. It then undergoes processing by the multi-head attention mechanism, followed by another residual join and layer normalization. Immediately after the input to the feed-forward layer, it finally undergoes residual concatenation and layer normalization to obtain the output of the T_De part.

In Path2, the input H first passes through the T_En1 section, yielding the result $Y_{p2}\_T\_En1$, and then the result is fed into the En_Multi_CNN part. En_Multi_CNN uses different sizes of convolution kernels and connections to process the one-dimensional sequences passing through T_En1. Unlike the convolution in the Path1 path, after the T_En1 operation, the data dimensions are changed. To better learn the features of the data in this one dimension, the one-dimensional convolution operation is used.

**Figure 5:**
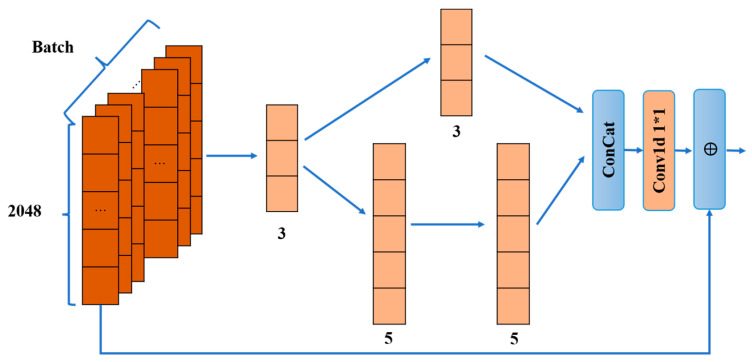
En_Multi_CNN. ‘*’ denotes the convolution dimension hyphen, e.g., 1*1 denotes a 1D kernel of size 1 × 1; ‘⨁’ indicates the addition of two matrices.

The details of the En_Multi_CNN section are shown in Figure 5, where one-dimensional convolution is used for all convolutions:

(20) $$Y_{1D}=Conv1D(Y_{in1d},W_{in1d})$$

Here, $Y_{in1d}$ denotes the sequence of inputs and $W_{in1d}$ denotes the weight matrix of the inputs, with dimensions determined by the input and output channels. Path2 first goes through a convolution of one dimension, with convolution size 3. In order to learn the features of different sizes more fully, it is divided into two paths; the first path directly adopts a one-dimensional convolution of size 3, which reduces the parameters while learning small-size features quickly and improving the efficiency of local and fine-grained feature learning, and obtains $Y_{p2}\_E1$. The second path uses two one-dimensional convolutions of size 5, and the two convolutions are concatenated together to learn features with larger sensory fields to help edge feature learning, obtaining $Y_{p2}\_E2$. Here, all the convolutions are computed as shown in Equation (20). After each convolution operation, one-dimensional batch normalization is performed:

(21) $${Y_{1D}}_{norm}=(\frac{{Y_{1D}}_{i}-mean\left({Y_{1D}}_{i}\right)}{sqrt\left(var(Y_{1D}\_i)+ep\right)})*g_{1d}+b_{1d}$$

Here, the $Y_{1D}\_i$ denotes the input sequence and $mean\left({Y_{1D}}_{i}\right)$ denotes the mean of the feature sequence, $var(Y_{1D}\_i)$ denotes the variance of the feature sequence, ep is a tiny constant used to avoid division by zero, and $g_{1d}$ and $b_{1d}$ denote the scaling factor and bias term. Then, after batch normalization, the nonlinearity is obtained using the LeakyReLU activation function. The outputs of the two trails are then Concat spliced:

(22) $$Y_{p2}\_E=Concat(Y_{p2}\_E1∥Y_{p2}\_E2)$$

“∥” denotes sequence splicing. Subsequently, a one-dimensional convolution operation of size 1 is accessed as in Equation (20), followed by a one-shot batch normalization and LeakyReLU activation function to reduce the network parameters. Finally, the input to En_Multi_CNN concerning the output of En_Multi_CNN with reference to the idea of residual connection:

(23) $${Y_{p2}\_E}^{’}=F2\left(Y_{p2}\_E\right)+Y_{p2}\_T\_En1$$

$F2\left(\cdot \right)$ denotes the sequence of operations to obtain $Y_{p2}\_E$. Then, ${Y_{p2}\_E}^{’}$ is fed to T_En2, which is processed in the same way as T_En1 to obtain ${Y_{p2}\_E}^{’’}$, and the nonlinearity is introduced using the Relu activation function. Finally, ${Y_{p2}\_E}^{’’}$ is passed into EnFc_2, which is computed as in Equation (13), according to the compression ratio η, to obtain υ2.

**Figure 6:**
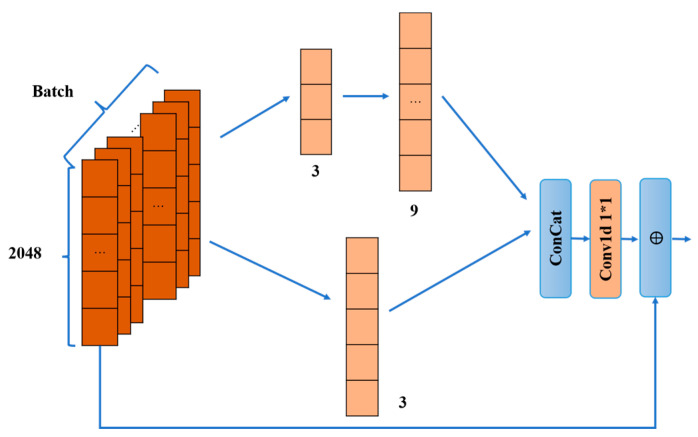
De_Multi_CNN. ‘*’ denotes the convolution dimension hyphen, e.g., 1*1 denotes a 1D kernel of size 1 × 1; ‘⨁’ indicates the addition of two matrices.

υ2 is transmitted to the Path2_D path through the ideal feedback link. υ2 is first restored to the pre-compression dimension calculation as in Equation (15) through DeFc_2 to obtain ${\upsilon 2}_{D}$. Then, ${\upsilon 2}_{D}$ is used as an input to get $Y_{p2}\_T\_De$ after T_De processing, and it is used as an input to De_Multi_CNN1. The structure of De_Multi_CNN1 and De_Multi_CNN2 is shown in Figure 6. Two different paths are used, and the two paths have different sizes and convolutions. The first path comprises a one-dimensional convolution of size 3. The second path is composed of a one-dimensional convolution of size 3 and a one-dimensional convolution of size 9 in series. Each convolution is computed as in Equation (20), followed by batch normalization as in Equation (21), and the activation function LeakyReLU is used to provide nonlinearity. Then, the two path features are subjected to Concat operation as in Equation (21), and, finally, the network parameters are reduced by a one-dimensional convolution operation of size 1. The convolution is formulated as in Equation (20). After convolution, the batch normalization is performed as in Equation (21), and, finally, $Y_{p2}\_De\_M$ is obtained. At the end of De_Multi_CNN1, $Y_{p2}\_T\_De$ and $Y_{p2}\_De\_M$ are summed up using residual join ideas. Calculated as in Equation (22) and using the Relu activation function to provide nonlinearity, the calculation yields ${Y_{p2}\_D}^{’}$. Then, it is processed by De_Multi_CNN2 with the same process as De_Multi_CNN1 to obtain ${\upsilon 2}^{’}$.

### 3.4. Mix_Multi_TransNet Network Outputs

After the processing of the two paths Path1 and Path2, the output is obtained, ${\upsilon 1}^{’}$ and ${\upsilon 2}^{’}$. Finally, ${\upsilon 1}^{’}$ and ${\upsilon 2}^{’}$ are fused:

(24) $$\hat{H_{a}}={\upsilon 1}^{’}⨁{\upsilon 2}^{’}$$

“⨁” denotes the addition of the two matrices, which ultimately results in the recovery of the channel matrix $\hat{H_{a}}$

### 3.5. Mix_Multi_TransNet Steps

The flow of the Mix_Multi_TransNet algorithm is shown in Algorithm 1. The input is H and the output is $\hat{H_{a}}$. Initialize the transmit antenna $N_{t}=32$, subcarrier $N_{c}=1024$, and then truncate the first Na = 32 rows. Firstly, we obtain H1 after a two-dimensional discrete Fourier variation, and then, according to the initialized truncation row $N_{a}$, we truncate $H^{’}$ to get $H_{a}$. Then, we split the complex matrix $H_{a}$ into a real part, $Re\left(H_{a}\right)$, and an imaginary part, $Im\left(H_{a}\right)$, and then the real and fictional elements are combined to form a new real matrix H, and we feed H to our encoder and decoder. H is provided to Path1 and finally gets ${\upsilon 1}^{’}$; see Section 3.2 Path1 for detailed steps. At the same time of feeding H to Path1, it is also fed to Path2 and finally gets ${\upsilon 2}^{’}$, see Section 3.3 Path2 for exact steps. Finally, after Equation (24), we obtain the recovered matrix $\hat{H_{a}}$.
**Algorithm 1:** Mix_Multi_TransNet Steps1Input: $H\epsilon C^{N_{c}\times N_{t}}$
2Output: $\hat{H_{a}}\epsilon C^{N_{c}\times N_{t}}$
3Initialize: $N_{t}=32$$,\;N_{c}=1024$$,\;N_{a}=32$
4$H^{’}=F_{c}HF_{t}^{H}$$,\;H^{’}\epsilon C^{N_{c}\times N_{t}}$$,\;F_{c}\epsilon C^{N_{c}\times N_{c}}$$,\;F_{t}\epsilon C^{N_{t}\times N_{t}}$5$\mathrm{Truncate}\;\mathrm{the}\;\mathrm{first}\;N_{a}$$\;\mathrm{rows}:\;H_{a}\epsilon C^{N_{a}\times N_{t}}$6$Re\left(H_{a}\right)=a\;;Im\left(H_{a}\right)=b$7$H_{\left[k\right][j]}=\left\{\begin{array}{c} Re\left({H_{a}}_{\left[i\right][j]}\right),0\leq k<N_{a}\\ Im\left({H_{a}}_{\left[i\right][j]}\right),N_{a}\leq k<2N_{a} \end{array},\right.0\leq i<Na,0\leq j<Nt,0\leq k<2Na$, i=k or i=k−Na8Path1: Path1_E, Path1_D9 Path1_E: υ1; Path1_D: ${\upsilon 1}^{’}$
10Path2: Path2_E, Path2_D11 Path1_E: υ2; Path1_D: ${\upsilon 2}^{’}$
12$\hat{H_{a}}={\upsilon 1}^{’}⨁{\upsilon 2}^{’}$13End

## 4. Simulation Results and Analysis

This section describes the detailed setup of the experiment and the network performance and compares the network accuracy with the state-of-the-art CSI feedback algorithm.

### 4.1. Data Sets, Training Programs, and Assessment Indicators

This study generates a dataset using COST2100 [29], and the proposed Mix_Multi_TransNet is compared with the existing state-of-the-art algorithms CsiNet, CsiNet+, CLNet, CRNet, and STNet. Two scenarios are considered in generating the dataset: an indoor scenario at 5.3 GHz and an outdoor scenario at 300 MHz, with a uniform linear array (ULA) model with $N_{t}=32$ at the BS. For FDD systems, $N_{c}=1024$ subcarriers are taken in the frequency domain. After two-dimensional Discrete Fourier Transform (DFT), $N_{a}=32$ is taken in the angular delay domain. It is divided into two scenarios, each with 150,000 independently generated channels, divided into training, validation, and test datasets containing 100,000, 30,000 and 20,000 channel matrices, respectively. The experiments split the data into individual matrix data for data loading convenience.

This experiment was conducted in Windows Server 2019 Standard environment using NVIDIA Quadro RTX 5000 Graphics Processing Unit (GPU) with 16 GB of video memory, 128 GB of RAM, and a Central Processing Unit (CPU) of i9-10900K clocked at 3.7 GHz for network training. The network is implemented based on Pytorch, and the Adam optimizer is used to train the network with 100 epochs, and the batch is set to 16. The learning rate is set to 0.001, and the learning rate is adjusted using the cosine annealing method, and the formula is calculated as follows:

(25) $$lr=l_{n}+\frac{1}{2}(l_{str}-l_{n})(1+\cos\left(\frac{T_{cur}}{T_{max}}\pi \right))$$

where lr denotes the current learning rate, $l_{n}$ denotes the final value after the learning rate has decayed, $l_{str}$ denotes the initial value of the learning rate, $T_{cur}$ denotes the current epoch value, and $T_{max}$ denotes the total epoch value.

In this thesis, the normalized mean square error (NMSE) between $H_{a}$ and $\hat{H_{a}}$ is used as a criterion for the network accuracy, which is calculated as given by Equation (26):

(26) $$NMSE=E(\frac{{\left\|H_{a}-\hat{H_{a}}\right\|}_{2}^{2}}{{\left\|H_{a}\right\|}_{2}^{2}})$$

### 4.2. Mix_Multi_TransNet Network Performance

Comparison details with existing CSI feedback algorithms are shown in Table 1. After 100 training rounds, Mix_Multi_TransNet starts to outperform other deep learning algorithms in both indoor and outdoor scenes. In the indoor scenario, the compression ratio η values equal to 1/8, 1/16, 1/32, and 1/64 outperform the other algorithms. They are compared with the best accuracy of the other algorithms, and Mix_Multi_TransNet obtains NMSE gains of 4.06 dB, 4.92 dB, 4.82 dB, and 6.47 dB, respectively. This means that in the indoor scenario, with compression ratio values of η=1/8, 1/16, 1/32, 1/64, the accuracy is improved by 49.59%, 64.70%, 65.60%, and 32.24%, respectively, compared with the optimal accuracy of the existing algorithm.

In the outdoor scene, all the compression ratio η values outperform the other algorithms, and Mix_Multi_TransNet obtains NMSE gains of 3.63 dB, 6.24 dB, 4.71 dB, 4.60 dB, and 2.93 dB, respectively. This implies that in the indoor scene, the accuracy improves compared to the optimal accuracy of the existing algorithms by 46.30%, 76.80%, 63.03%, 64.16%, and 50.04%, respectively.

In the bottom half of Table 1 is our network’s training time and each Batch’s response time when tested. The units are minutes and milliseconds, respectively.

**Figure 7:**
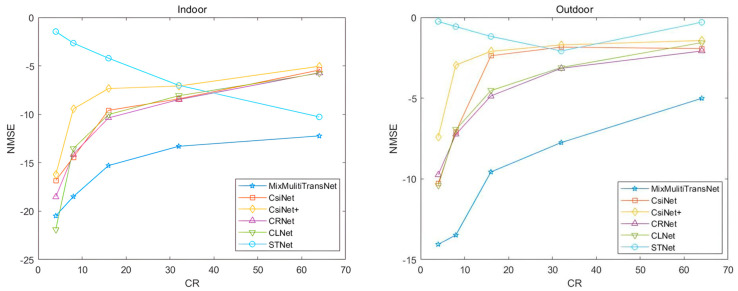
Performance comparison with other algorithms in Indoor and Outdoor.

As shown in Figure 7, in the indoor scenario, all compression ratio cases outperform the other accuracies, except for the compression ratio value η=1/4 when the network performance is slightly worse than CLNet. In the outdoor scenario, all compression ratio values outperform the other algorithms. The overall network performance shows a decreasing trend with increasing compression ratio values; the more significant the compression ratio value, the more the loss of channel state information and, after feedback, the accuracy of recovering the channel state information decreases. In outdoor scenarios, the advantage of the Mix_Multi_TransNet algorithm is more significant than other algorithms, which can maximize feedback accuracy and is more suitable for complex outdoor environments.

### 4.3. Ablation Experiment

As shown in Table 2, this study compares the network’s performance status when the network uses specific modules alone. Because this network uses dual paths, the spatial and temporal features of the channel state information are learned separately; finally, the matrix information of the two paths is fused, which improves the problem of low accuracy when training and learning from one aspect alone. Path1 focuses on learning the spatial features of the channel state information, while Path2 focuses on learning the temporal features of the channel state information. In Path2, the one-dimensional convolutional modules En_Multi_CNN, De_Multi_CNN1, and De_Multi_CNN1 are also used for more excellent compression. As obtained from Table 2, the network performance is not optimal when a particular path is used alone or in some of these modules.

In the indoor scene, using Path1 alone reduces the average NMSE gain by 2.10 dB compared to using the Path1 + Path2 no-convolution module and reduces the average NMSE gain by 7.61 dB compared to using the Path1 + Path2 complete network NMSE. This implies that in the indoor scene, using Path1 alone reduces the average NMSE gain by 53.18% compared to using the Path1 + Path2 no-convolution module The average NMSE accuracy is reduced by 53.18% and the average NMSE gain is reduced by 80.00% using the Path1 + Path2 complete network. Using Path2 alone in the indoor scene reduces the average gain by 2.31 dB over the Path1 + Path2 convolution-free module NMSE and 7.81 dB over the Path1 + Path2 full network NMSE, which implies that the average accuracy of Path2 alone in the indoor scene is reduced by 50.77% over the Path1 + Path2 convolution-free module NMSE. The average accuracy is reduced by 50.77% and the average gain is reduced by 79.05% compared to using the Path1 + Path2 complete network NMSE.

In the outdoor scene, the average NMSE gain of Path1 alone is 0.738 dB lower than that of the Path1 + Path2 no-convolution module and 5.51 dB lower than that of the Path1 + Path2 full network NMSE, which implies that the average NMSE accuracy of the outdoor scene is 14.58% lower than that of the Path1 + Path2 no-convolution module, and 78.42% lower than the Path1 + Path2 complete network NMSE. The average NMSE accuracy is reduced by 14.58%, and the average NMSE gain is reduced by 78.42% when using the Path1 + Path2 complete network. In the outdoor scene, using Path2 alone reduces the average gain by 2.39 dB over the Path1 + Path2 convolution-free module NMSE. It reduces the average gain by 7.16 dB over the Path1 + Path2 complete network NMSE, which means that in the outdoor scene, the average accuracy of Path2 alone reduces by 42.42% over the Path1 + Path2 convolution-free module NMSE. The average accuracy is reduced by 42.42%, and the average gain is reduced by 85.59% compared to using the Path1 + Path2 complete network NMSE.

## 5. Conclusions

This thesis proposes a multi-source neural network, Mix_Multi_TransNet, to solve the CSI feedback problem. The network learns different channel state information features and the two proposed paths learn spatial and temporal features, respectively, and obtain the encoder compression results of the channel state information matrix. The encoder compresses the channel state information matrix. Then, it is decoded by the decoder and the highest precision of the matrix is restored to the original channel state information matrix by fusing the feature information of the two paths. The Normalized Mean Square Error (NMSE) is used as an error measure and compared with existing algorithms from the COST2100 dataset. In the indoor scene, Mix_Multi_TransNet obtained the highest accuracy for compression ratios η=1/8, 1/16, 1/32, 1/64, and the NMSE gain was 4.06 dB, 4.92 dB, 4.82 dB, and 6.47 dB, respectively. Mix_Multi_TransNet obtained the highest accuracy in the outdoor scene for all compression ratio values n. In the outdoor stage, all the compression ratio values η, Mix_Multi_TransNet have the highest accuracy, and the gain obtained by NMSE was 3.63 dB, 6.24 dB, 4.71 dB, 4.60 dB, and 2.93 dB, respectively.

In this study, a significant gain in NMSE was achieved in the indoor and outdoor scenarios compared to other algorithms. In future work, we will focus more on improving the model’s inference speed and the model’s size for use in natural industrial environments.

## Figures and Tables

**Table 1:** NMSE Comparison between Mix_Multi_TransNet and Other Methods.

η	1/4		1/8		1/16		1/32		1/64	
Methods	NMSE									
	In	Out	In	Out	In	Out	In	Out	In	Out
Mix_Multi_TransNet	**−20.48**	**−14.05**	**−18.49**	**−13.48**	**−15.29**	**−9.57**	**−13.30**	**−7.75**	**−12.23**	**−5.01**
CsiNet	−16.83	−10.28	−14.43	−7.08	−9.59	−2.37	−8.41	−1.84	−5.43	−1.93
CsiNet+	−16.22	−7.40	−9.45	−2.95	−7.34	−2.10	−7.08	−1.70	−5.04	−1.43
CRNet	−18.51	−9.74	−14.12	−7.24	−10.37	−4.86	−8.48	−3.15	−5.71	−2.08
CLNet	−21.88	−10.42	−13.54	−6.95	−10.03	−4.53	−8.08	−3.10	−5.76	−1.56
STNet	−1.46	−0.25	−2.65	−0.57	−4.21	−1.18	−7.01	−2.08	−10.27	−0.30
Training Time(minutes)										
Mix_Multi_TransNet	259.16	264.07	257.14	264.41	256.26	263.81	257.50	265.93	260.86	264.80
	Batch’s Response Time (milliseconds)									
	12.27	12.24	12.23	12.21	12.24	12.23	12.24	12.25	12.19	12.18

η: Compression Ratio Value; In: indoor Scene; Out: outdoor Scene. Training Time (minutes) and Batch’s Response Time (milliseconds). The table below corresponds to “In” and “Out” above.

**Table 2:** NMSE comparison of ablation study.

η	Path1		Path2		Path1 + Path2 without Convolution		Path1 + Path2	
	In	Out	In	Out	In	Out	In	Out
1/4	−13.34	−8.77	−8.32	−2.90	−15.67	−9.66	−20.48	−14.05
1/8	−10.39	−5.92	−10.70	−2.62	−13.40	−6.84	−18.49	−13.48
1/16	−8.33	−3.46	−8.58	−1.29	−10.35	−4.30	−15.29	−9.57
1/32	−5.45	−2.21	−6.16	−2.92	−7.08	−2.72	−13.30	−7.75
1/64	−4.25	−1.95	−6.95	−4.30	−5.76	−2.48	−12.23	−5.01

*η*: Compression Ratio Value; In: indoor Scene; Out: outdoor Scene.

## Data Availability

The channel state information (CSI) matrix is generated from COST2100 model. You can also generate your dataset according to the open-source library of COST2100. The details of data preprocessing can be found in our paper. COST2100 open source libraries address link: https://github.com/cost2100/cost2100.
